# Effect of source and journey on physiological variables in calves transported by road and ferry between Ireland and the Netherlands

**DOI:** 10.3389/fvets.2023.1238734

**Published:** 2023-09-26

**Authors:** Luca L. van Dijk, Susanne Siegmann, Niamh L. Field, Katie Sugrue, Cornelis G. van Reenen, Eddie A. M. Bokkers, Gearoid Sayers, Muireann Conneely

**Affiliations:** ^1^Teagasc, Animal and Grassland Research and Innovation Centre, Fermoy, Ireland; ^2^Department of Biological and Pharmaceutical Sciences, Munster Technological University, Tralee, Ireland; ^3^Animal Production Systems Group, Wageningen University and Research, Wageningen, Netherlands

**Keywords:** health, calf, welfare, stress, fasting, long-distance transport, livestock export

## Abstract

This study aimed to establish baseline variables for calves transported by road and ferry from Ireland to the Netherlands and to investigate the effect of journey [two comparable journeys in April (J1) and May (J2) 2022] and source [source farm or mart (SF/MA)] on these variables. A total of 66 calves from the SF/MA were transported from Ireland to commercial veal farms in the Netherlands. Blood samples were collected at the SF/MA, assembly center (Ireland), lairage (France), and on arrival on the veal farm (Netherlands). They were analyzed for indicator variables related to energy balance, hydration/electrolytes, physical/muscular stress, immunity, and inflammation [glucose, beta-hydroxybutyrate (BHB), non-esterified fatty acids (NEFA), potassium, sodium, magnesium, chloride, urea, haematocrit, total protein, creatine kinase, L-lactate, cortisol, white blood cell, neutrophil, lymphocyte and monocyte counts, serum amyloid-A, and haptoglobin]. Health variables eye and nose discharge, skin tent (a measure of dehydration), and navel inflammation were scored by a trained observer, and calves were weighed at every blood-sampling time point. All blood variables and body weight changed significantly (*P* < 0.05) during transport, most notably between the assembly center and lairage. Reference ranges were available for 18 variables; 11 of these variables exceeded the reference ranges at the lairage, whilst 10 variables exceeded the reference ranges on arrival at the veal farm. However, health variables did not change during transport. A journey-to-journey comparison indicated much variation; 18 out of 25 variables differed significantly on at least one time point. In total, J1 calves experienced a more severe change in BHB, potassium, strong-ion-difference, L-lactate, and eye and nose discharge than J2 calves. The source of calves also affected their physiology; 12 out of 25 variables studied differed significantly, all of which were confined to the first time point. Specifically, MA calves had elevated levels of NEFA, urea, haematocrit, L-lactate, cortisol, white blood cell, neutrophil, and monocyte counts and lower levels of corrected chloride and lymphocyte count. Overall, calves in this study showed a generalized physiological disturbance beyond reference limits during long-distance transport, but no animal died during transport or for 3 weeks post-arrival.

## 1. Introduction

Globally, the transport of young calves is a critical component of the dairy industry. Dairy cows have a calf annually to produce milk; surplus calves, in particular males, which cannot be used to produce milk, are typically raised for veal or beef or sold for early slaughter and must be transported to reach the rearing facility or slaughterhouse (1). In Ireland, a proportion of these “surplus” calves are transported to mainland Europe for this purpose. The transport of pre-weaned calves can have significant impacts on their health, welfare, and productivity, as well as on the economic viability of the dairy and veal industries (1, 2). Calves are typically worth more when sold for veal or beef than for early slaughter, and the veal industry depends on calves arriving at veal farms in a healthy state, emphasizing the need for proper care and management during transportation. Male dairy calves destined for rearing facilities or early slaughter can be transported as early as a few days of age in the USA, Australia, and New Zealand (3) or between 2 and 4 weeks of age in EU member states and the UK (4). These calves are most susceptible to disease between 2 and 4 weeks of age when their immune system is transitioning from maternal to calf antibodies (5, 6). The additional stressors of transport (including adverse climatic conditions, fasting, and the commingling of calves from many different farms) result in further immunosuppression (7), leading to the invasion of pathogens into the respiratory and digestive tracts. Additionally, young calves rely on milk for the majority of their nutrition, as their gastrointestinal tract is not fully developed for digesting solids (8). In the EU, calves must be fed a suitable amount of milk (or milk replacer) at least twice a day when housed (Council Directive 2008/119/EC). Regulations do not state the amount of milk (replacer) that must be fed, but calves are commonly fed 3 L of milk or milk replacer twice a day in Ireland (9). Energy demands may be higher during transport due to stress and constant readjustments to overcome the truck's movements and are compounded by infrequent feeding (10, 11). In the EU, unweaned calves must be given a rest period of at least 1 h after 9 h of travel, in particular for them to be given liquid and if necessary fed, after a further 9 h of transport, they must be unloaded and fed and watered (Council Regulation (EC) No 1/2005). A derogation does exist which allows for exemptions to these rules for trade with remote member states.

Each year, more than 120,000 mostly male calves of <6 weeks of age are exported from Ireland to other EU member states (12), of which approximately 46,000 are transported by road and ferry to veal farms in the Netherlands [2020 and 2021 (12)]. Irish calves destined for the EU are transported directly from their farm of birth or via a commercial livestock mart (Mart; MA) to an assembly center, where they are fed in advance of transport by road and ferry to France. In the current system between Ireland and mainland Europe, calves remain on the livestock lorry for the duration of the ferry crossing, typically 18 h, and as of yet, no systems that allow for the feeding of milk replacer on the lorries are commercially available. On arrival in France, calves are unloaded and fed at a control post (also referred to as “lairage”) near the port; they remain at the lairage for 13 h, before continuing the journey to a commercial veal farm in the Netherlands or rearing facilities in other EU member states.

Irish calves destined for European veal or beef production are transported intermittently for several days, between the dairy farm, assembly center, lairage, and destination farm. The transport between Ireland and continental Europe is also seasonal; most calves are transported between February and April, and the journey can be greatly influenced by weather. Temperature, wind, and wave height can influence the transport duration and the environment inside the truck. Therefore, different consignments of calves in different “journeys” may experience various impacts on their health and welfare. Extended transport durations and long stays in assembly centers are associated with hypoglycaemia (11). Even when glucose levels are not low enough to be clinically defined as hypoglycaemia, calves experience a negative energy balance; this can be associated with feelings of hunger, which is a drive to maintain homeostasis (13). Negative energy balance and increases in levels of non-esterified fatty acids (NEFA) and beta-hydroxybutyrate (BHB) have been observed in calves in relation to feed withdrawal and transport (14, 15).

The combination of a compromised immunity with feed withdrawal during transport affects the health and welfare of calves. Transport (18–20 h) has been shown to negatively alter hematology (16) and biochemistry (11), and even short transport (2h) was shown to increase cortisol levels (17). The transport between Ireland and Europe, however, is different from road-only transport in that its overall transport duration is longer and encompasses longer feed withdrawals and a compulsory ferry journey. The effect of this unique combination of a road/ferry journey on young calves may affect the blood composition of calves differently compared with shorter journeys. No research has yet been performed on unweaned calves transported from Ireland to continental Europe. There is no baseline of variables that indicates the current state of health and welfare during the transport of these calves, which is vital to identify and implement changes that will improve the wellbeing of these transported calves.

This observational study aimed to establish baseline blood variables of unweaned dairy-bred calves during transport from Ireland to veal farms in the Netherlands. Furthermore, we aimed to assess the effect of the source of the calf (e.g., directly from the dairy farm or via the livestock MA), as well as the effect of two transports on different dates (journeys) on blood variables in calves during this transport.

## 2. Materials and methods

### 2.1. Calf selection

This trial was performed during two commercial shipments of calves between Ireland and the Netherlands in April (J1) and May (J2) 2022 using calves collected at source farms (SFs) or commercial livestock MAs. SFs were selected by the livestock exporter based on the availability of at least two calves for export for the set departure date. All healthy Holstein-Friesian and Holstein-Friesian x beef calves over 14 days of age, regardless of sex and breed, that were presented for transport at the SFs or purchased at the MA, were included.

For J1, a total of 17 calves (all males; average age 30.6 days, five Friesian, nine Friesian x Aberdeen Angus, and three Friesian x Hereford) from four SFs (all based in County Cork, Ireland) were included in the study. An additional 20 MA calves (all males; average age 29.2 days, 14 Friesian x Aberdeen Angus and six Friesian x Hereford) sourced from a commercial MA (County Cork, Ireland) were also enrolled.

For J2, a total of 10 calves (six male; average age 32.3 days, four female and five male Friesian x Aberdeen Angus and one male Friesian x Hereford) from four SFs (all based in County Cork, Ireland) were included in the study. In addition, 19 MA calves (14 male; average age 26.6 days, 10 male Friesian, five female, and four male Friesian x Aberdeen Angus) sourced from a commercial MA (County Cork, Ireland) were also enrolled.

All calves arrived at the exporter's assembly center (County Wicklow, Ireland) the night before ferry transport, and the estimated transport times between the SF/MA and assembly center were 6 to 12 h.

### 2.2. Transport

**Figure 1:**
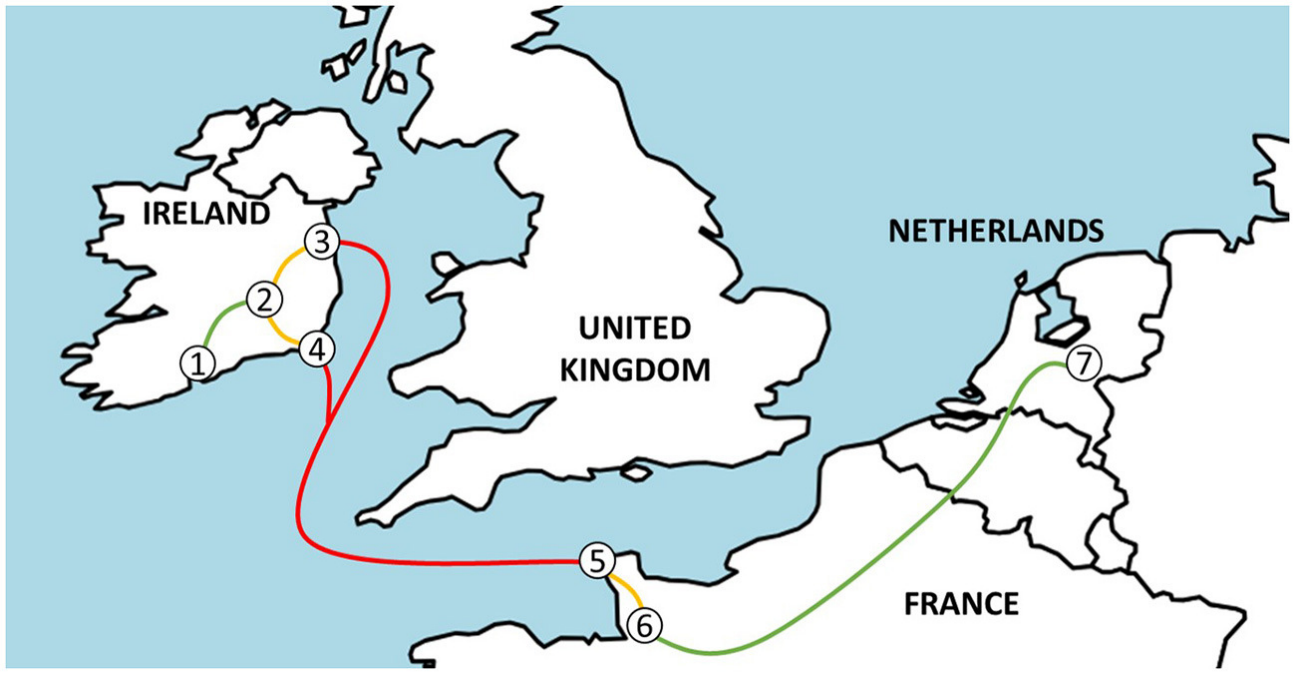
A map indicating the journey from Ireland to the Netherlands from source farms (1), via the assembly center (2) and Dublin (3) or Rosslare (4) port in Ireland, Cherbourg port (5), and the lairage (6) in France to veal farms in the Netherlands (7).

**Figure 2:**
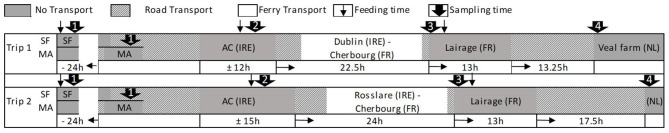
Schematic display of transport times, including feeding and sampling times and approximate journey durations for journey 1 and journey 2 during transport from the calves' origin (1) via the assembly center (2) and the lairage (3) to the veal farm (4).

A display of transport routes is shown in Figure 1. A schematic display including transport durations, sampling, and feeding times is presented in Figure 2. For J1, SFs were visited and calves were sampled 1 day before they were picked up by the exporter (T ≈ −24 h; SF). MA calves arrived at the MA the next morning (T ≈ −4 h) and were sorted and sold through the sale ring before sampling (T = 0 h; MA). SF and MA calves were transported to the assembly center the same day and arrived at T ≈ 10 h (the exact transport times were not recorded; the estimated transport times were 6— 12 h). Calves were offloaded and penned on deeply bedded straw overnight. They were loaded onto the livestock lorry at T ≈ 22 h and departed within 1 h. The livestock lorry boarded the ferry at Dublin port at T ≈ 26 h; during the ferry journey, the livestock lorry was parked on an outside deck, and calves were exposed to prevailing weather. The weather was mild with a slight overcast (15.8°C, humidity: 72%; average data recorded by two TinyTags positioned on the inside wall in the front and rear of the lorry), and the sea was calm with minimal swell [mean wave height of 1.6 m at M5 buoy; (18)]. The ferry arrived in Cherbourg, France at T ≈ 43 h. A 1-h transport to Tollevast in France followed, where calves were offloaded at the lairage at T ≈ 44 h. Calves were penned on deeply bedded straw overnight and were re-loaded onto the livestock lorry at T ≈ 57 h. The livestock lorry arrived at the veal farm in Markelo, the Netherlands at T ≈ 70 h. Between France and the Netherlands, the weather was mild with a slight overcast (15.9°C, humidity: 67%).

For J2, transport conditions were similar, but calves departed the assembly center at T ≈ 25 h and boarded the ferry at Rosslare port at T ≈ 31 h. The ferry arrived in Cherbourg, France at T ≈ 49 h, and calves were offloaded at the lairage at T ≈ 50 h. They were re-loaded onto the livestock lorry at T ≈ 63 h and arrived at the veal farm in Eibergen, the Netherlands at T ≈ 80 h. During the ferry journey, the weather was mild with a slight overcast (17.7°C, humidity: 66%; average data recorded by two TinyTags positioned in proximity to the lorry), and the sea was rough [mean wave height of 3.6 m at M5 buoy; (18)]. Between France and the Netherlands, there were heavy downpours (20.3°C, humidity: 82%; average data recorded by two TinyTags positioned on the inside wall in the front and rear of the lorry).

### 2.3. Sampling and feeding

A schematic display of transport, sampling, and feeding times is shown in Figure 2. For every sampling timepoint, calves were weighed (loaded onto calibrated scales), blood sampled (26 ml was taken by jugular venepuncture by experienced personnel; 20G needle), and health scored. On all SFs, calves were fed whole milk or milk replacer by the farmer according to their own protocols approximately 6 to 10 h before sampling (SF/MA); however, no feeding information was available for MA-sourced calves. At the assembly center, calves were fed 2 L of milk replacer [Pro calf milk replacer (Wicklow Calf Company, Ireland); 21% protein, 17% fat] ~2 h before sampling (assembly center). At the lairage, calves were fed 3 L of milk replacer [Denkamilk Top milk (Denkavit, the Netherlands); 22% protein, 19% fat] after sampling (lairage). In both cases, feed buckets with individual compartments were used, but feeding was not monitored by the trial staff. At the veal farm, calves were fed an electrolyte mix in water after sampling (veal farm).

Clinical health scores included various variables. Scoring protocols were based on the Wisconsin calf health scorer (19) and adapted for functionality in transport research. For the purpose of this study, all clinical health scores were transformed to a binary value to assess “healthy” and “not healthy” calves. Calves were scored with eye discharge when (at minimum) a small amount of unilateral ocular discharge was present. A nose score was recorded when (at minimum) a small amount of unilateral cloudy discharge was recorded. A navel score was recorded when the navel was mildly inflamed and measured approximately the width of a pointer finger or more, was hot, or the calf gave a pain response (reacted to touching of the navel by kicking or moving away intentionally). Calves were scored with a positive skin tent when the skin tent, after pinching, did not return to normal within 1 s. All observers received official training for clinical health scoring from a veterinarian in advance of the trial. Observer one scored all calves at the SF, MA, assembly center, and lairage, whilst observer one or two took clinical health scores on arrival at the veal farm.

For each journey between Ireland and France, as well as between France and the Netherlands, absolute temperature and relative humidity were measured inside and outside the livestock lorry using TinyTags (Gemini Data Loggers Ltd., UK).

### 2.4. Laboratory analysis

At each time point, four blood samples per calf were collected in four different tubes containing 6 ml EDTA, 6 ml heparin, 6 ml glycolytic inhibitor, and 8.5 ml serum separator tubes (SST). EDTA tubes were refrigerated, and hematology (haematocrit and white blood cell, neutrophil, lymphocyte, and monocyte counts) was analyzed using the Advia 2120 system (Bayer, AG) at Teagasc Grange (SF/MA and assembly center samples; Dunsany, Ireland) or using fluorescence flow cytometry (XT-1800i, Sysmex Europe GmbH, Germany) at Rimondia (lairage and veal farm samples; Elspeet, the Netherlands).

All remaining samples were spun at 3,000 rpm for 5 min (heparin and glycolytic inhibitors) or 10 min (SST), decanted into serum tubes, and stored in a freezer (−20°C). All serum tubes were sent to Teagasc Grange (Dunsany, Ireland) for biochemistry (glucose, BHB, NEFA, sodium, calcium, urea, total protein, L-lactate, creatine kinase, and haptoglobin) analysis using a colourmetric AU 480 chemistry analyser (Beckman Coulter, USA), except for magnesium, potassium, and chloride, which were analyzed using the ISE AU 480 chemistry analyser (Beckman Coulter, USA). ELISA was used to analyse cortisol (Enzo AD1-901-071, Brussels, Belgium) and serum amyloid-A (Tri Delta TP802, Maynooth, Ireland).

### 2.5. Statistical analysis

Prior to statistical analysis, one measurement for BHB and NEFA showed a negative value, which was deemed the laboratory analysis's fault and thus removed. Electrolyte values (sodium, potassium, and chloride) for four calves were deemed too low to be correct and were eliminated from the dataset. Strong ion difference (SID) was calculated based on the formula [SID = Na + K – Cl].

All statistical analyses were performed in SAS On Demand (20). Repeated measures data of glucose, potassium, sodium, chloride, corrected chloride, magnesium, haematocrit, total protein, SID, and body weight were analyzed with a linear mixed model (SAS procedure PROC MIXED). Variables for which residuals failed normality tests upon initial analysis were analyzed using a generalized linear mixed model (SAS procedure PROC GLIMMIX). These variables were analyzed using a lognormal distribution (BHB, creatine kinase, cortisol, L-lactate, serum amyloid-A, haptoglobin, white blood cell, neutrophil, lymphocyte, and monocyte counts). When lognormal transformations did not sufficiently normalize data, the square root of the variable was used (NEFA, urea). Health variables were analyzed using a binary distribution with a logit link function (skin tent, eye discharge, nose discharge, and navel inflammation). The covariance structure was decided based on the AIC fit statistic [ARH (1), AR (1), CSH, or Unstructured]. Residuals were checked visually for normality using a Q-Q plot and by plotting residuals against predicted values.

For both the linear mixed model and the generalized linear mixed model, the following model was used:


$$\begin{array}{l} \mu +{\text{Time}}_{\text{i}}+{\text{Journey}}_{\text{j}}+{\text{Source}}_{\text{k}}+{\text{Breed}}_{\text{l}}+{\text{Age}}_{\text{m}}\\ +{\text{WeightStart}}_{\text{n}}+\left({\text{Time}}_{\text{i}}\times {\text{Journey}}_{\text{j}}\right)+\left({\text{Time}}_{\text{i}}\times \;{\text{Source}}_{\text{k}}\right), \end{array}$$

where μ is the population mean, and Time_i_ (I = SF/MA, assembly center, lairage, and veal farm), Journey_j_ [j = April (1), May (2)], Source_k_ (k = SF, MA), and Breed_l_ (l = HF, AAX, HEX) are fixed effects. The model also included interactions between time and journey as well as between time and source. Age_n_ (*n* = age at departure, 16 d−42 d), and WeightStart_m_ (*n* = Weight at SF or MA, 43–72 kg) were included as continuous covariates. Interactions were considered significant when a *P* < 0.05. Non-significant interactions were manually removed from the model in a step-wise fashion (those with the highest *P*-values were removed first) to improve the power of the remaining variables. Least squares means were used to assess pairwise comparisons between time and journey and between time and source when relevant interactions were significant (*P* < 0.05). The model was run a second time without including the interactive effects of time and journey and time and source to assess the main effect of Time; a *post-hoc* test (Tukey) was conducted to adjust for multiple comparisons in the least squares means of this analysis. Model estimates, confidence limits, and standard errors (where applicable) from log- and square root-transformed data were back-transformed (exponential and squared, respectively) for use in figures and tables. Raw data were used to present binary data.

### 2.4. Ethical approval

The experiment was approved by the Teagasc Animal Ethics Committee (Fermoy, Ireland, Approval number: TAEC2021-326) and the Health Products Regulatory Authority (Dublin, Ireland, Approval number: AE19132/P154).

## 3. Results

All calves that commenced the international section of the journey reached the veal farms in the Netherlands, and veterinary care was not sought for any animal at any stage of the journey or on arrival.

### 3.1. Changes in physiology over time

Mean values of all physiological variables per time point, significant changes over time, and reference ranges published in previous research are shown in Table 1. Published reference ranges were observed in healthy male and female dairy or dairy cross calves between birth and 4 weeks of age (21–24, 26) or between 1 and 12 months of age [L-lactate; (25)].

**Table 1:** Estimated means (SE) (pooled by journey and source) of baseline physiological and health parameters during transport of young calves (n = 66) at four locations between Ireland and the Netherlands.

	**Source farm/mart**	**Assembly center**	**Lairage**	**Veal farm**	***P*-value**	**Reference range**	
Glucose (mmol/L)	5.02 ± 0.15^a^	6.24 ± 0.17^b^	3.39 ± 0.09^c^	3.55 ± 0.07^c^	< 0.0001	3.9–8.4	(21)
Beta-hydroxy butyrate (mmol/L)	0.11 ± 0.06^a^	0.1 ± 0.08^a^	0.34 ± 0.06^b^	0.37 ± 0.05^b^	< 0.0001	0–0.13	(22)
Non-esterified fatty acids (mmol/L)	0.2 ± 0.01^a^	0.21 ± 0.02^a^	0.62 ± 0.02^b^	0.65 ± 0.02^b^	< 0.0001	0.04–0.27	(23)
Potassium (mmol/L)	5.53 ± 0.06^a^	5.28 ± 0.07^b^	6.9 ± 0.1^c^	5.67 ± 0.06^a^	< 0.0001	4.13–5.41	(21)
Sodium (mmol/L)	140.4 ± 0.27^a^	141.3 ± 0.27^b^	144.6 ± 0.26^c^	142.9 ± 0.3^d^	< 0.0001	133.3–140.2	(21)
Magnesium (mmol/L)	0.66 ± 0.01^a^	0.72 ± 0.02^bc^	0.68 ± 0.01^ab^	0.73 ± 0.01^c^	< 0.0001		
Corrected chloride (mmol/L)	95.4 ± 0.26^a^	96.5 ± 0.24^b^	96.2 ± 0.23^c^	98.5 ± 0.17^d^	< 0.0001		
Strong-ion difference (mmol/L)	48 ± 0.26^a^	46.9 ± 0.27^b^	49.8 ± 0.29^c^	45.7 ± 0.21^d^	< 0.0001	39.4–48.8	(21)
Urea (mmol/L)	1.87 ± 0.06^a^	3.29 ± 0.05^b^	3.51 ± 0.05^bc^	3.68 ± 0.05^c^	< 0.0001	0.4–3.4	(22)
Haematocrit (%)	32.81 ± 0.82^a^	31.45 ± 0.86^b^	37.73 ± 0.96^c^	34.24 ± 0.9^d^	< 0.0001	25–43	(21)
Total protein (g/L)	58.4 ± 0.72^a^	60.4 ± 0.79^b^	56.4 ± 1.1^a^	60 ± 0.81^b^	< 0.0001	40–90	(24)
Creatine kinase (u/L)	155 ± 0.07^a^	177.3 ± 0.06^a^	185.2 ± 0.06^a^	225.4 ± 0.06^b^	< 0.0001	90–120	(22)
L-lactate (mmol/L)	2.14 ± 0.03^a^	1.54 ± 0.06^b^	1.47 ± 0.05^c^	1.76 ± 0.03^d^	< 0.0001	0.33–1.37	(25)
Cortisol (ng/ml)	8.55 ± 0.12^a^	10.85 ± 0.11^b^	14 ± 0.09^c^	10.37 ± 0.11^d^	< 0.0001	0–2	(22)
White blood cell count (10^9^/L)	10.3 ± 0.04^a^	10.8 ± 0.06^a^	9.6 ± 0.04^a^	8.2 ± 0.04^b^	< 0.0001	2.55–23.73	(24)
Neutrophil count (10^9^/L)	3.83 ± 0.07^a^	4.37 ± 0.1^a^	3.93 ± 0.06^a^	2.96 ± 0.07^b^	< 0.0001	0.38–15.78	(24)
Lymphocyte count (10^9^/L)	4.09 ± 0.07^a^	4.7 ± 0.05^b^	3.53 ± 0.05^c^	3.49 ± 0.05^d^	< 0.0001	0.17–9.45	(24)
Monocyte count (10^9^/L)	0.82 ± 0.06^a^	0.79 ± 0.06^a^	1.48 ± 0.05^b^	1.15 ± 0.05^c^	< 0.0001	0.01–2.11	(24)
Serum amyloid-A (mg/L)	59.3 ± 0.12^a^	75.3 ± 0.1^b^	81.3 ± 0.11^b^	76.8 ± 0.09^ab^	0.006	< 0.178	(26)
Haptoglobin (mg/ml)	1.68 ± 0.06^a^	1.8 ± 0.06^b^	1.24 ± 0.07^c^	1.71 ± 0.06^ab^	< 0.0001	0.07–0.46	(23)
Skin tent (% of calves)	1.59 ± 1.59^ab^	8.93 ± 3.85^ab^	21.21 ± 5.07^a^	41.54 ± 6.16^c^	< 0.0001		
Eye discharge (% of calves)	18.2 ± 4.78	37.5 ± 6.53	22.7 ± 5.20	31.8 ± 5.78	0.0766		
Nose discharge (% of calves)	3.0 ± 2.13	0 ± 0	0 ± 0	16.7 ± 4.62	0.1552		
Navel inflammation (% of calves)	28.8 ± 5.62	33.9 ± 6.38	40.9 ± 6.10	33.3 ± 5.85	0.4972		
Body weight (kg)	55.9 ± 0.17^a^	55.6 ± 0.18^b^	52.3 ± 0.17^c^	53.4 ± 0.17^d^	< 0.0001		

*P*-value reflects the main effect of time between the four sampling locations. Reference ranges are taken from previously published research.

Mean values without a common superscript (^a, b, c, d^) differ significantly (P < 0.05).

Variables that are indicators for energy balance changed over time; glucose was the lowest at the lairage and veal farm, where 74% and 68% of calves, respectively, fell below the lower reference limit. BHB and NEFA were low at the SF/MA and the assembly center but increased between the assembly center and lairage and remained high thereafter; the upper reference limits were exceeded by more than 96% of calves at the lairage and veal farm.

Electrolytes potassium, sodium, and corrected chloride showed an increase over time. At the lairage, 100% of calves exceeded the upper reference limits for potassium and sodium. Changes in magnesium and SID over time were variable, although SID increased between the assembly center and lairage and decreased between the lairage and veal farm.

The hydration indicator variable urea showed an increase over time as the duration of transport increased; the upper reference limits were exceeded by some calves at all time points but most frequently at the veal farm, where 68% of calves had urea concentrations above the upper reference limit. Haematocrit and total protein showed the respective highest and lowest values at the lairage, but the reference limits were rarely exceeded, most often for haematocrit at the lairage, where 23% of calves had levels above the upper reference limit. Skin tent showed the greatest increase over time, most notably between the lairage and veal farm, where more than 41% of calves showed dehydration based on skin tent.

In terms of variables that are related to muscle fatigue/stress, creatine kinase was the highest on arrival at the veal farm, whilst L-lactate was the highest at the SF/MA. Changes in cortisol over time were variable. The upper reference limits were exceeded by more than 50% of calves at all time points but most frequently at the lairage (cortisol: 100% of calves) or veal farm (L-lactate: 89%, creatine kinase: 96% of calves).

All variables that are related to immunity status showed a general decrease during transport, except for monocyte count, which increased. The upper and lower reference limits were rarely exceeded. Monocyte count exceeded the upper reference limit most often, notably so at the lairage (12% of calves).

For inflammation, serum amyloid-A increased between the SF/MA and the assembly center but remained similar thereafter. Haptoglobin was the lowest at the lairage but showed a stabilizing pattern at the veal farm. The upper reference limit for serum amyloid-A was rarely exceeded, most frequently at the lairage (15% of calves), whilst the upper reference limit for haptoglobin was exceeded by more than 96% of calves at all time points.

There was minimal evidence that health scores changed significantly over time (*P*-values: eye discharge = 0.0766, nose discharge = 0.1552, and navel inflammation = 0.4972). Body weight changed between all time points, the largest decrease in body weight was seen between the assembly center and lairage. Body weight increased slightly between the lairage and veal farm but remained lower than that before transport.

### 3.2. Effect of journey

**Figure 3:**
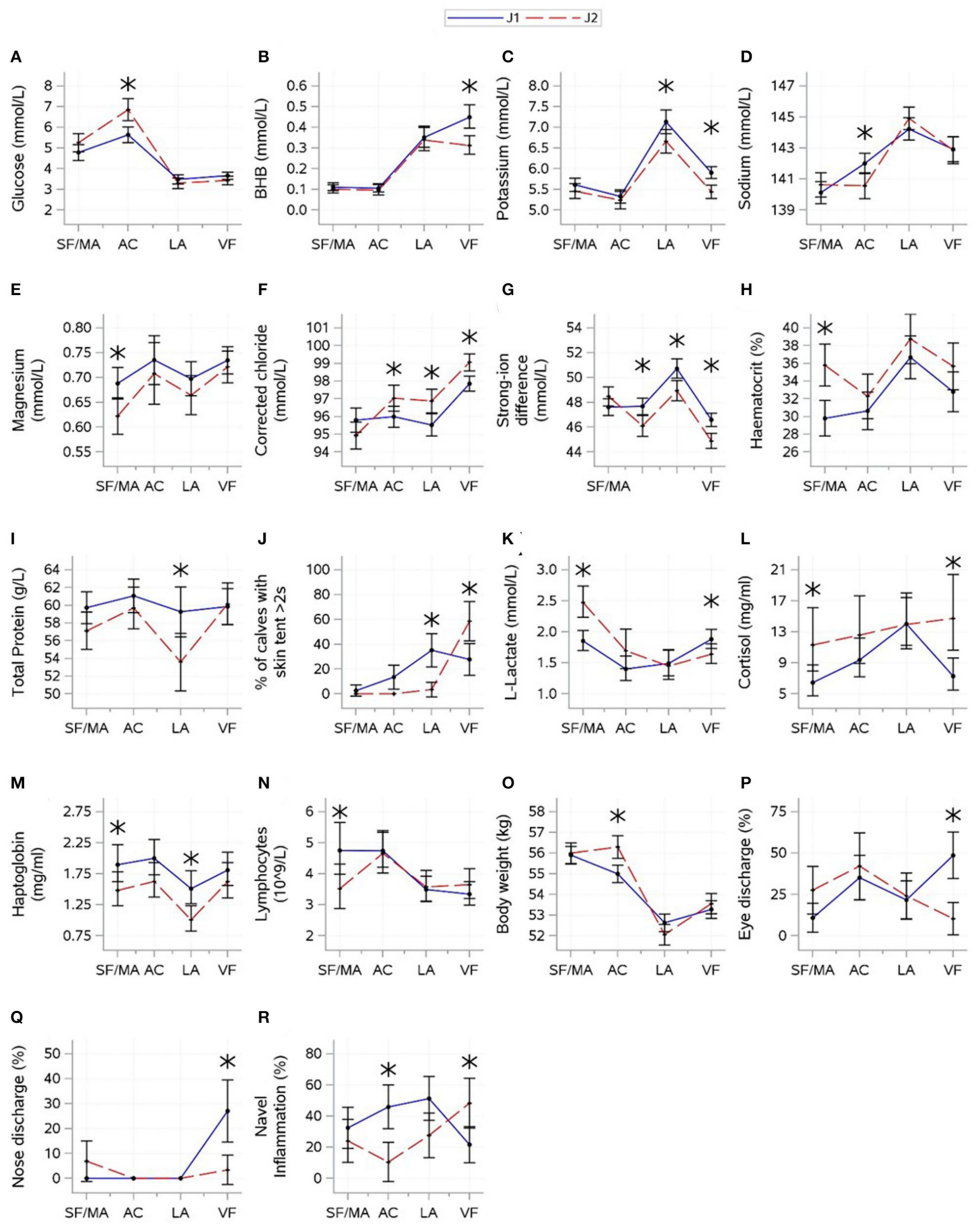
Interactions (mean ± CI) between journey (April and May 2022) and time [source farm/mart (SF/MA), assembly center (AC), lairage (LA), and veal farm (VF)]. Mean (SE) values for glucose **(A)**, beta-hydroxybutyrate [BHB **(B)**], potassium **(C)**, sodium **(D)**, magnesium **(E)**, corrected chloride **(F)**, strong-ion difference **(G)**, haematocrit **(H)**, total protein **(I)**, L-lactate **(J)**, cortisol **(K)**, skin tent **(L)**, haptoglobin **(M)**, lymphocytes **(N)**, body weight **(O)**, eye discharge **(P)**, nose discharge **(Q)**, and navel inflammation **(R)** are shown. Asterisks indicate significant difference between journeys (*P* < 0.05) within a sampling moment.

The results for interactions between journey and time are shown in Figure 3. For indicator variables of energy balance, an interaction between journey and time was found for glucose (*P* = 0.0004) and BHB (*P* = 0.0047). Glucose and BHB showed roughly opposing patterns. Glucose increased between the SF/MA and assembly center, where J2 had higher glucose. A drop was observed thereafter, but the levels remained similar between groups. BHB increased between the assembly center and lairage, and J1 continued to increase until the veal farm, where the journeys were different. There was minimal evidence that the journey significantly affected NEFA (*P* = 0.4001).

For electrolyte variables, an interaction between journey and time was found for all variables (*P*-values: potassium = 0.0230, sodium = 0.0018, magnesium = 0.0202, corrected chloride = 0.0002, and SID < 0.0001). J1 had higher potassium at the lairage and veal farm but lower sodium at the assembly center. SID was the lowest, and corrected chloride was the highest at the veal farm. J1 had higher corrected chloride but lower SID at the assembly center, lairage, and veal farm than J2. Magnesium was variable over time and showed no consistent changes, but J1 had higher magnesium at the SF/MA.

For hydration-related variables, an interaction between journey and time was found for haematocrit (*P* < 0.0001), total protein (*P* = 0.0003), and skin tent (*P* = 0.0049). J1 had lower haematocrit at the SF/MA but lower total protein at the lairage; differences between journeys were not found at other times. Skin tent was the highest at the lairage for J1 but peaked at the veal farm for J2. There was no evidence that journeys significantly differed in the levels of urea (*P* = 0.8431).

For variables related to muscle fatigue/stress, an interaction between journey and time was found for L-lactate (*P* < 0.0001) and cortisol (*P* = 0.0007). A decrease in L-lactate between the SF/MA and any subsequent time points was found for J2, whilst this change was not as pronounced for J1. J1 had lower L-lactate at the SF/MA and higher L-lactate at the veal farm. Cortisol was generally higher for J2 (although only significantly at the SF/MA and veal farm), but standard deviations for cortisol within J2 were large. The overall mean values of creatine kinase differed between journeys (*P* = 0.0041; J1: 173 ± 9.12, J2: 223 ± 11.1).

For immunity-related variables, an interaction between journey and time was found for lymphocyte count (*P* = 0.0102). J2 started off with lower lymphocyte counts at the SF/MA, but changes in lymphocyte count over time were similar between journeys thereafter. There was no evidence that the overall means of WBC, neutrophil count, and monocyte count significantly differed between journeys (*P* > 0.05).

For inflammation-related variables, an interaction between journey and time was found for haptoglobin (*P* = 0.0007). Haptoglobin values were higher for J1 than for J2 calves at the SF/MA and lairage. There was no evidence that the overall mean values of serum amyloid-A significantly differed between journeys (*P* = 0.8463).

For health scores and weight, an interaction between journey and time was found for all variables (*P*-values: eye discharge = 0.0033, nose discharge = 0.0011, navel inflammation = 0.0017, and weight < 0.0001). Mean body weight was higher for J2 at the assembly center, but this was not significant at any other time. For J1, frequencies of eye discharge and nose discharge were the highest at the veal farm; for J2, eye discharge was the lowest at the veal farm, and nose discharge did not change over time. Journeys showed roughly opposite patterns for navel inflammation, and differences in the prevalence of navel inflammation were found at the assembly center, where J1 had a higher prevalence of navel inflammation, and veal farm, where J2 showed a higher prevalence.

### 3.3. Effect of source

**Figure 4:**
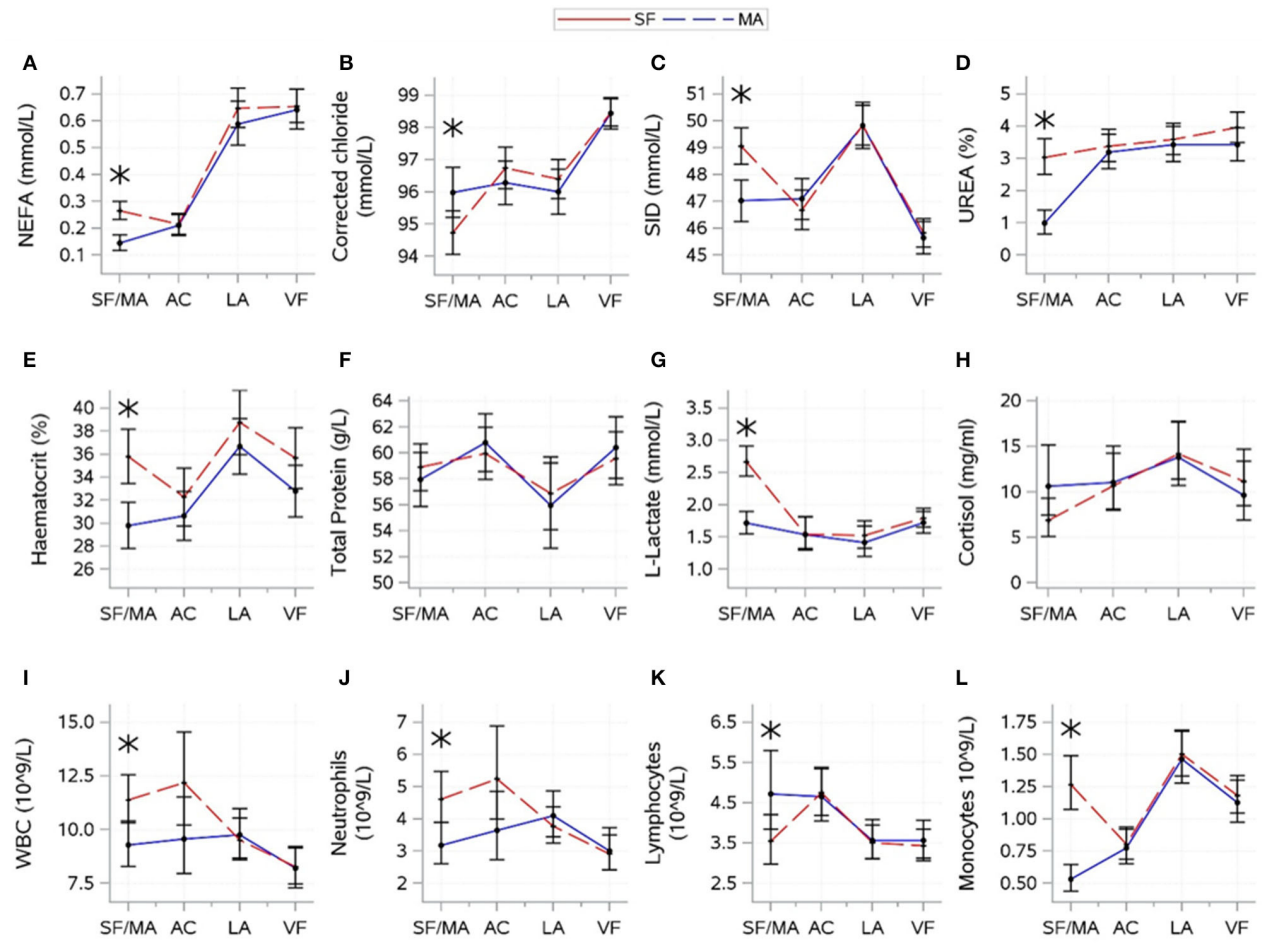
Interactions (mean ± CI), between source [source farm or mart (SF/MA)] and time [(SF/MA), assembly center (AC), lairage (LA), and veal farm (VF)]. Mean (SE) values of NEFA **(A)**, corrected chloride **(B)**, strong-ion difference **(C)**, urea **(D)**, haematocrit **(E)**, total protein **(F)**, lactate **(G)**, cortisol **(H)**, WBC **(I)**, neutrophils **(J)**, lymphocytes **(K)**, and monocytes **(L)** are shown. Asterisks indicate significant difference between sources (*P* < 0.05) within a sampling moment.

The results for interactions between source and time are shown in Figure 4. For variables indicative of energy balance, an interaction between source and time was found for NEFA (*P* = 0.0007). NEFA was higher for MA calves at the SF/MA, but sources showed similar patterns thereafter. There was no evidence of a significant difference between sources in glucose (*P* = 0.7378) and BHB (*P* = 0.9572).

For electrolyte-related variables, an interaction between source and time was found for corrected chloride (*P* = 0.0029) and SID (*P* = 0.0005). SID and corrected chloride were lower for SF than for MA at the SF/MA. Sources (overall mean values) tended to differ in potassium (*P* = 0.0660; SF: 5.45 ± 0.06, MA: 5.65 ± 0.06) and sodium (*P* = 0.0760; SF: 142.3 ± 0.18, MA: 142.8 ± 0.16), but there was no evidence that source significantly affected magnesium (*P* = 0.8006).

For hydration-related variables, an interaction between source and time was found for urea (*P* < 0.0001), haematocrit (*P* = 0.0034), and total protein (*P* = 0.0409). Urea and haematocrit were higher for MA calves at the SF/MA, but sources showed similar patterns thereafter. Total protein was variable over time, and sources did not differ at any one time point. There was no evidence that source significantly affected skin tent (*P* = 0.9108).

For variables related to muscle fatigue/stress, an interaction between source and time was found for L-lactate (*P* < 0.0001) and cortisol (*P* = 0.0409). L-lactate was high for MA calves at the SF/MA, which differed from SF calves. L-lactate remained low for both sources thereafter. Changes in cortisol over time were variable, and sources did not differ at any time. There was no evidence that the overall mean values of creatine kinase significantly differed between sources (*P* = 0.1845).

For immunity-related variables, a difference between source and time was found for all variables (*P*-values: WBC = 0.0044, neutrophils = 0.0034, lymphocytes = 0.0058, and monocytes < 0.0001). WBC, neutrophil, and monocyte count were higher for J2 at the SF/MA, but the lymphocyte count for J2 was lower at this time. Sources did not differ in any immunity-related variable thereafter.

For health scores and weight, there were no significant interactions between source and time for any variable. The overall means of the two sources were different for navel inflammation (*P* = 0.0061; SF: 42.6 ± 4.78, MA: 28.1 ± 3.73) and tended to differ for eye discharge (*P* = 0.0717; SF: 6.5 ± 4.52, MA: 4.1 ± 3.51). There was no evidence that the overall means for nose discharge (*P* = 0.2194) and body weight (*P* = 0.1432) were significantly affected by the source of the calves.

## 4. Discussion

This observational study aimed to record and comparatively assess the impact of transport of Irish calves involving both sea and road, across two journeys and from two commercial sources. The current study confirmed that long-distance transport has a declining effect on the physiological status of calves with a generalized and progressive deterioration of several blood and health variables in calves over time, relative to physiological norms. It must be noted that all calves arrived at their destination, and no calves died for at least 3 weeks post-transport. Regardless, these results reinforce conclusions drawn from previously published research data for calves transported over land for short and long durations (11, 16, 27), although these studies did not include a combination of ferry and lorry transport.

Overall, mean values for 11 out of 18 variables (for which reference ranges were available) exceeded the reference ranges published by previous studies after long fasting periods (21–26). Journey had a significant effect on blood variables in transported calves; 18 out of 25 variables differed between journeys on at least one time point. Source affected 12 of the 25 variables, however significant variations were found exclusively at the SF/MA, indicating the effect of the origins of the animals (in particular the transport/handling at the MA for MA calves) but one that was corrected after feeding at the assembly center.

### 4.1. Changes in physiology over time

Perhaps being the most obvious effect in this study, calves showed a negative energy balance at the lairage and veal farm. A negative energy balance has been reported following long fasting durations and transport (11, 14–16), as demonstrated by a decrease in glucose and an increase in BHB and NEFA. Falling below the lower reference limit in the case of glucose is not uncommon in these situations, and in this study, it was confirmed to have occurred both at the lairage and at the veal farm. Glucose is a tightly regulated blood metabolite; its concentration is determined by the interplay between insulin and glucagon, the balance of which serves to prevent glucose fluctuations. It is evident from this study that a physiological base limit for energy balance was reached following the first long fasting period [to the lairage, where 15.4% of calves were hypoglycaemic (Glucose < 2.8 mmol/L)], as there was minimal change in any of the three metabolites between the lairage and the final destination (10.8% of calves hypoglycaemic) despite another period of transport and fasting.

In this study, electrolytes and other hydration-related variables showed an increase in concentrations beyond the upper reference limit at the lairage or veal farm (potassium, sodium, and urea); the percentage of calves with a positive skin tent also increased. Due to transport and prolonged fasting, a decrease in the total blood volume and resulting dehydration could be expected (28). Electrolytes and total protein have been shown to increase in response to long-distance transport (11, 16, 29), although not all previous research found differences in potassium, sodium, magnesium, total protein, or urea after transport exceeding 18 h (14–16, 30). Such a profile points to a decline in the total plasma volume; in the case of sodium and urea, this can be attributed to vasoconstriction and increased reabsorption by the kidneys (31, 32) and/or an increase in protein catabolism (33). A physiological balancing act could also account for some of these changes. Elevated levels of anions (BHB and lactate) will decrease the pH, which can be stabilized through an increase in SID (mainly driven by sodium and potassium) and a potential concurrent decrease in protein.

In contrast to findings in previous research (11, 30), neither total protein nor haematocrit concentrations varied outside the reference ranges throughout the journey, although an incremental increase in haematocrit was found between the SF/MA and lairage, which likely reflected the lack of water/milk intake during the transport. These conflicting results in hydration variables may be indicative of the early stages of a physiological response to dehydration whereby shifts in electrolytes can be used to balance fluid compartments (blood volume, in particular), thereby accommodating reduced intake of fluids and maintaining homeostasis. The fact that the external scoring of hydration (i.e., skin tent) is not always directly related to other methods of measuring dehydration (34, 35) may indicate a priority toward preserving blood volume over subcutaneous tissues and that skin tent is likely an indicator of early-stage dehydration. In this study, a positive skin tent was evident in 1% of calves at the start of the journey, 20% of calves at the lairage, and 40% of calves at the veal farm. This variable is sensitive to observer bias; however, an overall profile of the declining hydration status of calves was evident in this study. The average decline in weight between the SF/MA and lairage was 3.7 kg (6.6% of body weight), which can be partially accounted for by fluid loss. Calves can lose between 1% and 7% of body weight during transport lasting up to 19 h (15, 16), and body weight loss in the current study fell within these limits. Body weight loss due to transport and fasting of this magnitude poses the question of whether access to water during transport should be improved.

Cortisol and lactate levels in this study, as indicators of (muscle) stress, were above the reference limit but showed inconsistent changes during the journey. Both cortisol and lactate have been shown to increase in response to transport in calves (11, 17, 30), whilst in other research, creatine kinase and lactate were not affected by transport but did increase in response to stocking density or exposure to prevailing weather (11, 15, 36). Cortisol levels in the current study were low compared to those of other studies (17, 37); however, the short half-life of this glucocorticoid affects the results. For example, in calves, cortisol can return to normal levels within 80 min after a stressful event that increases cortisol by 300% (38). Blood samples in the present study were collected within 2 h of arrival at the lairage and veal farm, allowing cortisol levels to return to normal levels; therefore, they may not be a good representation of transport stress. Creatine kinase values were comparable to those of previous research (11) and showed a similar accumulative increased pattern between the assembly center and veal farm. The highest values were recorded at the veal farm following the road section of the journey where animals may have strained more against the movements of the truck than during the ferry section of the journey.

There was no evidence of significant shifts in immune (white blood cell, neutrophil, and lymphocyte counts) or inflammatory variables. With the exception of haptoglobin, the mean values of all variables in these two variable groups remained within reference ranges, despite some minor but significant changes over time. In parallel to this, health scores (eye discharge, nose discharge, and navel inflammation) did not change during transport, even though visual observations hinted at an increase in eye and nose discharge throughout the study. It must be noted that a single observer performed all health scores throughout the study, except for the veal farm, where there were two observers; the effect of this is unknown. The evidence from the markers of inflammation was ambiguous with serum amyloid-A showing no change over time and remaining within reference ranges, whilst haptoglobin levels were above the reference limit throughout. Transport, in most cases, involves the commingling of calves, and this poses a high risk for the spread of pathogens (39). Immune responses and associated clinical signs require time to respond (40), and the lack of a sizable response in this study could indicate a response in development or a simple reduction in infection potential for these calves. The observed small but significant decline in immune subsets over time may indicate migration to marginating pools, leading to an overall decrease in immune subsets in the blood. Monocytes showed a similar but delayed pattern, which was expected as monocyte count typically responds to stress and infection slower than other immune subsets (40). An acute phase response has been previously observed in calves coping with infection (41) or in response to acute stress (such as transport) (42).

The accumulative effects of transport over time prevent conclusions from being drawn on the individual effect of ferry (sea) transport vs. road transport on the physiology of calves. The physiological status of calves at the lairage (after the ferry journey) and veal farm (after road transport) are different in many cases (glucose, all electrolytes, haematocrit, total protein, creatine kinase, L-lactate, white blood cell, neutrophil, and monocyte count, haptoglobin, skin tent, and body weight), with the veal farm observations not always more negative, as one might expect, given the longer cumulative journey duration. Given Ireland's geographical constraints, a ferry journey is an unavoidable component of transporting Irish calves to mainland Europe. Elucidating the effect of a sea journey alone, and determining if this represents a critical point in their overall journey, is a future aim of this overall research.

### 4.2. Effect of journey

To our knowledge, little research has been conducted on the differences between multiple consignments transported on a similar journey. The potential for meaningful variations between journeys, even whilst taking a similar route, is extensive. In this study's results, the journey itself had a significant impact on the physiology of calves, as 18 out of 25 physiological variables showed interactive effects of the journey. Whilst legislation aims to control variations between journeys by enforcing transport and feeding regimes, age, and health of the animal, uncontrolled factors are also at play, *inter alia*, prevailing weather, handling at loading and unloading, delays, driver experience, position within a truck, and parked position on the ferry (exposed or sheltered). Previous studies have also reported that using a conditioned versus an open truck influenced immune cell subsets (43), but calves transported in summer and winter showed little difference in clinical variables after a 19-h journey (16). Calves with a low body weight were shown to respond to transport differently than calves that had a normal body weight and similar age (17). Distance has also been shown to affect calf physiology; negative energy balance and dehydration are exacerbated by transport duration (11).

In this study, journey 2 had a longer transport time (J1 = 70 h, J2 = 80 h), with more extremely prevailing weather conditions (rough sea and heavy downpours). However, unexpectedly, J1 calves were visibly worse on arrival (reflected in eye and nose discharge) and showed a more severe decline in BHB, potassium, SID, L-lactate, and eye and nose discharge than J2 calves. The only variable that was visibly exacerbated by the long transport duration in J2 was skin tent, which was recorded twice as often in the second journey on arrival at the veal farm. Whilst further studies are required, this analysis suggests that journey-to-journey variation is an important factor and plays a significant role in disturbing the physiological status of calves, but the transport conditions that cause this are unknown.

### 4.3. Effect of source

The source of calves, whether SF or MA, was a significant cause of variation in 12 of the variables examined. However, in all cases, this difference was only evident at the first time point (SF/MA). In contrast to the SF calves, the MA calves encountered an added trip from the SF to the MA, albeit of short duration, in addition to the associated handling at the livestock MA. Whilst it could be assumed that these MA calves were fed prior to departure from the farm (feeding times for these calves were unknown), the average NEFA value for this group was almost double that of the SF calves. Also, all indicators of immunity were different from those observed in SF calves. This observation, in addition to the fact that the average cortisol levels were also elevated, but not significantly so, in the MA cohort, is suggestive of an elevated stress and hunger profile in these calves prior to sampling at the SF/MA. This novel finding indicates that the source of calves affects their baseline levels.

However, whilst impacting the calves at the SF/MA, the effect of source in this study did not transcend beyond this time point, evidenced by the fact that source was not significant at any time point beyond the SF/MA. In fact, by the assembly center sampling, following transport to, and feeding at, the assembly center, both cohorts displayed comparable physiological results, a trend that continued for the remainder of the journey. The long-term impact of source was not a focus of this report; however, the cumulative effect of an additional feed withdrawal and transport of MA calves relative to SF calves requires analysis.

### 4.4. Limitations

At the start of the study, SF calves were sampled 1 day prior to commencing their transport journey, in contrast to MA calves who were sampled on the day. As this sample was taken as a baseline sample, it was not expected to change in a day as calves remained on their farm of birth. A greater limitation was the timing of sampling around feeding, particularly at the assembly center where sampling was conducted after feeding, unlike the other timepoints. Unfortunately, this feature could not be overcome on the day.

### 4.5. Conclusion

Overall, calves showed a decline in their physiological status during the journey between Ireland and the Netherlands; the changes were most obvious during the ferry journey between Ireland and France, and minimal changes were observed between the lairage in Cherbourg and the veal farm. The current dataset indicates that the calves sold and transported through a commercial livestock MA prior to international transport had physiological variables that were affected at the MA, but feeding at the assembly center seemed to “reset” these changes. Furthermore, differences between SF and MA calves were not found at other time points. There were definite differences between different consignments (journeys) of calves transported on a similar journey, although each journey affected the variables in different ways. A general agreement on calves that arrived in a worse state could not be concluded, despite a difference in transport time and conditions.

## Data availability statement

The raw data supporting the conclusions of this article will be made available by the authors, without undue reservation.

## Ethics statement

The animal studies were approved by Teagasc Animal Ethics Committee and the Health Products Regulatory Authority. The studies were conducted in accordance with the local legislation and institutional requirements. Written informed consent was obtained from the owners for the participation of their animals in this study.

## Author contributions

LD carried out trial work, data analysis, and writing of manuscript. SS carried out trial work. NF supervised during trial work and data analysis. KS, CR, and EB equally contributed to planning and assisting in trial work. GS assisted in data analysis and writing of manuscript. MC supervised during trial work. All authors contributed to the article and approved the submitted version.
